# Synthesis and Magnetic and Optical Properties of Novel Fe@ZSM-5 Composites

**DOI:** 10.3390/molecules31010089

**Published:** 2025-12-25

**Authors:** Irina A. Zvereva, Denis A. Pankratov, Elena G. Zemtsova, Vladimir K. Kudymov, Azamat Samadov, Sergey A. Kurnosenko, Sergey O. Kirichenko, Marina G. Shelyapina, Vitalii Petranovskii

**Affiliations:** 1 Institute of Chemistry, Saint Petersburg State University, 7/9 Universitetskaya Embankment, Saint Petersburg 199034, Russia; irina.zvereva@spbu.ru (I.A.Z.); e.zemtsova@spbu.ru (E.G.Z.); v.k.kudymov@spbu.ru (V.K.K.); st088712@student.spbu.ru (A.S.); s.kurnosenko@spbu.ru (S.A.K.); 2 Department of Chemistry, Lomonosov Moscow State University, Leninskie Gory 1/3, Moscow 119991, Russia; pankratov@radio.chem.msu.ru; 3 Moscow Center for Advanced Studies, 20, Kulakova Str., Moscow 123592, Russia; 4 Centre for Innovative Technologies of Composite Nanomaterials, Saint Petersburg State University, 7/9 Universitetskaya Embankment, Saint Petersburg 199034, Russia; sergey.kirichenko@spbu.ru; 5 Department of Nuclear Physics Research Methods, Faculty of Physics, Saint Petersburg State University, 7/9 Universitetskaya Embankment, Saint Petersburg 199034, Russia; 6 Centro de Nanociencias y Nanotecnología, Universidad Nacional Autónoma de México, Ensenada 22800, BC, Mexico; vitalii@ens.cnyn.unam.mx

**Keywords:** zeolite, ZSM-5, mesoporosity, magnetite, composite, Mössbauer spectroscopy, vibrating-sample magnetometry, UV-Vis spectroscopy

## Abstract

Alkaline treatment in 0.2 and 0.4 M NaOH solutions successfully generated controlled mesoporosity into ZSM-5 (Zeolite Socony Mobil-5) zeolite, resulting in average mesopore diameters of approximately 15 and 25 nm, respectively, while preserving the crystalline structure of the zeolite framework. Parent ZSM-5 and its mesoporous derivatives obtained by desilication were used to prepare (Fe species)@(zeolite matrix) composites. The synthesis was carried out by co-precipitating Fe^2+^/Fe^3+^ ions onto both parent and desilicated ZSM-5 matrices under oxygen-free conditions. Comprehensive characterization by X-ray diffraction, scanning electron microscopy, N_2_ adsorption, vibrating-sample magnetometry, ^57^Fe Mössbauer spectroscopy, and diffuse reflectance UV–Vis spectroscopy revealed that the degree of introduced mesoporosity dramatically influences the size, dispersion, phase composition, and oxidation state of the iron-containing nanospecies. On purely microporous ZSM-5, relatively large (~15 nm) partially oxidized magnetite nanoparticles are formed predominantly on the external surface, exhibiting superparamagnetism at room temperature (*Mₛ* = 11 emu/g) and a band gap of 2.12 eV. Increasing mesoporosity leads to progressively smaller and more highly dispersed iron(III) oxo/hydroxo clusters with significantly lower blocking temperatures and reduced magnetization (down to 0.7 emu/g for Fe@ZSM-5_0.4). All composites display strong visible-light absorption confirming their potential as magnetically separable visible-light-driven photocatalysts for environmental remediation.

## 1. Introduction

Zeolites have been among the most important heterogeneous catalysts [1,2] and adsorbents [3,4] for several decades due to their ordered microporous structure (pore diameters of 0.3–1.2 nm) [5], high specific surface area (typically 300–700 m^2^/g) [6], excellent thermal and chemical stability [7], and the ability to finely tune their Brønsted and Lewis acidity [8,9], as well as their cation composition [10,11]. In recent years, increasing attention has been devoted to their application in photocatalytic purification of water and air [12,13,14], where zeolites serve either as supports for photoactive species or as intrinsic wide-band-gap semiconductor materials [14,15,16,17]. However, pristine zeolite frameworks possess a large band gap (*E*_g_ > 3.0–3.8 eV), which restricts their photoactivity to the ultraviolet region of the spectrum—a region that constitutes less than 5% of the solar radiation reaching the Earth’s surface [18]. This severely limits their practical utilization in solar-driven photocatalysis and increases energy consumption when artificial UV sources are required [12].

A widely adopted strategy to overcome this limitation involves modification of zeolites with photoactive transition metals and their oxides, which narrow the effective band-gap, extend light absorption into the visible region, and create heterojunctions that facilitate efficient separation of photogenerated electrons and holes [14,19,20,21]. Among the various candidates, iron oxides (in particular magnetite Fe_3_O_4_, maghemite γ-Fe_2_O_3_, and hematite α-Fe_2_O_3_) attract the greatest attention due to their earth abundance, low cost, environmental compatibility, high photochemical stability, and suitable electronic band-edge positions [12,22,23]. Magnetite is especially attractive because of its relatively narrow band gap of about 2.2–2.4 eV, which enables strong absorption of visible light up to ~600 nm and facilitates multielectron charge-transfer processes [24]. Iron oxides are redox active [25,26]. Under both UV and visible irradiation, Fe_3_O_4_ efficiently generates highly reactive oxygen species, including hydroxyl radicals (•OH), superoxide anion radicals (O_2_•^−^), and singlet oxygen (^1^O_2_) [27].

A particularly important advantage of magnetite is its pronounced ferrimagnetic properties, with saturation magnetization of ~90–100 emu/g at room temperature [28]. This property allows rapid and nearly complete magnetic separation of catalyst nanoparticles from the treated aqueous suspensions using a simple external permanent magnet or a weak electromagnet. This addresses two critical technological challenges of heterogeneous photocatalysis—facile reuse of the catalyst and prevention of secondary contamination by metal-based nanoparticles [29,30,31].

Composites combining ZSM-5 zeolite with deposited or encapsulated magnetite nanoparticles (commonly denoted as Fe@ZSM-5 or Fe_3_O_4_@ZSM-5) exploit multiple synergistic effects: (i) the high adsorption capacity of the zeolite toward organic pollutants, which increases the local reactant concentration near the photoactive sites; (ii) high dispersion and firm anchoring of magnetite nanoparticles within the pores and on the external surface of the zeolite, suppressing their aggregation and leaching; (iii) the possibility of magnetic separation and multiple recycling without significant loss of activity; (iv) photoresponse, significantly extended into the visible spectral region. Altogether, these features make such materials highly promising heterogeneous photocatalysts for the degradation of a wide range of persistent organic pollutants, including phenols, chlorophenols, textile dyes (e.g., methylene blue, rhodamine B, Congo red), pharmaceutical residues, and pesticides in aqueous media [14,23,32,33,34].

The target properties of Fe@zeolite composites depend on both the characteristics of the zeolite matrix and the method used for the synthesis and introduction of iron species [23,35]. The most commonly employed techniques include wet impregnation [36,37], ion exchange [38,39,40] (including sonochemically assisted variants [41]), and hydrothermal synthesis [42,43].

Conventional 3D zeolites are classified as microporous materials, since the size of their voids (*d*) typically does not exceed 1 nm. Their highly ordered structures with precisely defined pores, whose diameters match the size of small molecules, endow crystalline zeolites with valuable shape- and size-selectivity. However, as a direct consequence of these very advantages, the micropores impose significant diffusion limitations on the mass transfer of bulkier molecules.

The prospect of extending zeolite applications to new fields has prompted numerous attempts to synthesize mesoporous zeolitic materials that combine diffusion pathways on multiple length scales [44]. Consequently, in recent years growing interest has focused on zeolites with hierarchical porosity [6,45,46,47,48,49,50,51,52,53], i.e., zeolitic materials that integrate micro-, meso- and/or macropores. According to the IUPAC classification, porous materials are categorized as microporous (*d* < 2 nm), mesoporous (2 < *d* < 50 nm), and macroporous (*d* > 50 nm). The introduction of secondary (meso- and/or macro-) porosity is generally regarded as a strategy to enhance mass transfer and accelerate reaction kinetics [6,44,45,54,55]. However, it also influences the nature, dispersion, and phase composition of the supported iron species [40].

Thus, the design and comprehensive investigation of novel composites based on zeolite matrices with varying degrees of post-synthetically generated mesoporosity, modified with magnetite, represent a highly relevant and promising research direction. Such materials aim to create efficient, cost-effective, and technologically viable photocatalytic systems to address pressing environmental challenges in modern water purification.

In this contribution we report on the synthesis of novel Fe@ZSM-5 magnetic composites and their comprehensive study by complementary methods providing access to structural, textural, magnetic and optic properties of systems with magnetic species dispersed on a zeolite support, focusing the attention on the role of mesoporosity of the support on the nature and phase composition of formed iron species.

## 2. Results and Discussion

### 2.1. XDR, Morphology and Elemental Analysis

According to the nomenclature accepted by the International Zeolite Association (IZA), ZSM-5 has the MFI framework type, which is a three-letter code system recommended by the IZA to identify different zeolite framework structures [5]. The X-ray diffraction (XRD) patterns as well as textural properties of the zeolite matrices used as supports for iron species were previously reported in Ref. [40]. It was shown that alkaline treatment (i) preserves the MFI framework structure, (ii) significantly enhances mesoporosity: the parent material is predominantly microporous, whereas in the treated samples mesopores dominate the total pore volume. The average mesopore diameters are approximately 15 and 23 nm for ZSM-5_0.2 and ZSM-5_0.4, respectively.

**Figure 1:**
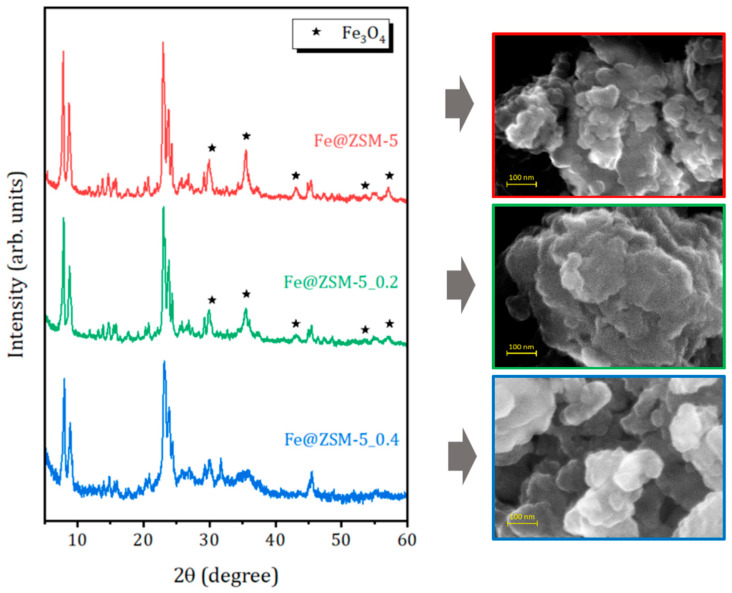
XRD patterns (on the **left**) and SEM images (on the **right**) of the studied composites. Solis symbols in the XDR patterns corresponds to iron Fe_3_O_4_ or γ-Fe_2_O_3_ oxide phases (see the text).

XRD patterns of the prepared composites are shown in Figure 1. All samples retain the characteristic reflections of the MFI structure. The patterns of Fe@ZSM-5 and Fe@ZSM-5_0.2 display additional diffraction peaks at 2θ = 30.4, 35.6, 43.1, 53.6 and 57.3°, which correspond to the (220), (311), (400), (422) and (511) planes of magnetite Fe_3_O_4_ (JCPDS # 00-065-0731) or maghemite γ-Fe_2_O_3_ (JCPDS # 84–1595). These two phases are difficult to distinguish solely by XRD due to their nearly identical patterns. In contrast, sample Fe@ZSM-5_0.4, prepared using the zeolite matrix subjected to more severe alkaline treatment, exhibits a broad amorphous halo in the 2θ range of 20–35° (typical of hierarchical zeolites [51,56]) and no discernible reflections attributable to Fe_3_O_4_.

The morphology of the composite sample particles was studied by SEM. As shown in Figure 1 and Figure S1 (Supplementary Materials), all samples consist of plate-like individual crystals approximately 100–200 nm in size, aggregated into micron-sized particles. The introduction of iron species does not significantly alter the overall morphology of the zeolite particles (see Figure S1).

The chemical composition of the parent and desilicated zeolite matrices was reported previously [40] and listed in Table 1. The results confirm partial silicon leaching from the framework after alkaline treatment and concomitant substitution of the original [NH_4_]^+^ ions with Na^+^.

The elemental distribution mappings obtained by energy-dispersive X-ray (EDX) spectroscopy reveals a uniform distribution of elements in the Fe@zeolite composites, see Figure S2 in Supplementary Materials. The results of the elemental analysis are summarized in Table 1.Incorporation of iron increases the Si/Al ratio, likely due to partial replacement of framework Al^3+^ by Fe^3+^. A substantial excess of Fe compared to Al confirm the formation of extra-framework iron-containing species, consistent with the XRD results (Figure 1). Notably, sample Fe@ZSM-5_0.4 exhibits the highest iron content, while shows no detectable crystalline iron oxide reflections in XRD, suggesting that the iron oxide nanoparticles are either highly dispersed or smaller than ~5 nm (below the XRD detection limit).

### 2.2. Surface Chemical Environment Analysis

**Figure 2:**
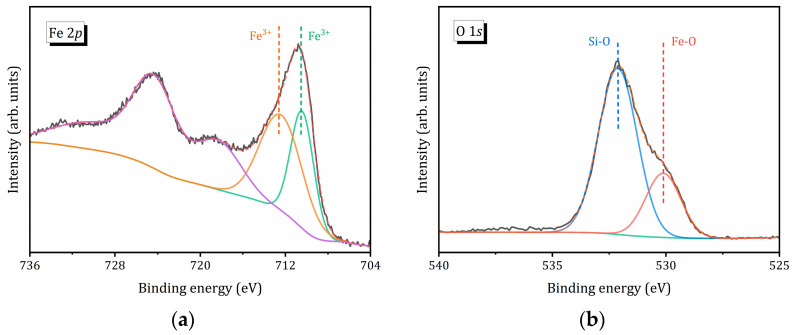
XPS spectra of Fe 2*p* (**a**) and O 1*s* (**b**) of the Fe@ZSM-5_0.4 composite. Colored lines show the decomposition of the corresponding XPS peaks into two Gaussian lines.

Figure 2 shows the Fe 2*p* and O 1*s* X-ray photoelectron spectroscopy (XPS) spectra of the Fe@ZSM-5_0.4 composite. The high-resolution Fe 2*p* (Figure 2a) displays main binding energy peaks at approximately 711 eV and 725 eV, corresponding Fe 2*p*_3/2_ and Fe 2*p*_1/2_, respectively [57,58]. A broad satellite peak appears at around 718 eV [58]. However, only the Fe 2*p*_3/2_ peak can be unambiguously deconvoluted into two Gaussian components at 710.5 eV and 712.6 eV, which can be assigned to Fe^2+^ and Fe^3+^ species, respectively. The Fe^2+^ fraction is calculated to be 40% in Fe@ZSM-5_0.4 (Table 2). Table 2 also shows that the Fe^2+^/Fe^3+^ ratio on the composite surface, as determined by XPS, slightly increases with increasing mesoporosity of the zeolite support.

Figure 2b illustrates the O 1*s* XPS spectrum of Fe@ZSM-5_0.4, which can be fitted with two Gaussian peaks located at 532.1 eV and 530.1 eV. The latter peak is attributed to oxygen associated with oxidized iron species (O_Fe_) [58], whereas the peak at 532.1 eV is related to zeolite framework oxygen (O_Z_) [17]. As shown in Table 1 and Table 2, with increasing mesoporosity of the zeolite support, the iron content increases; however, the relative contribution of O_Fe_ on the surface decreases. This observation suggests that iron species are preferentially formed inside the mesopores.

### 2.3. N_2_ Adsorption/Desorption

**Figure 3:**
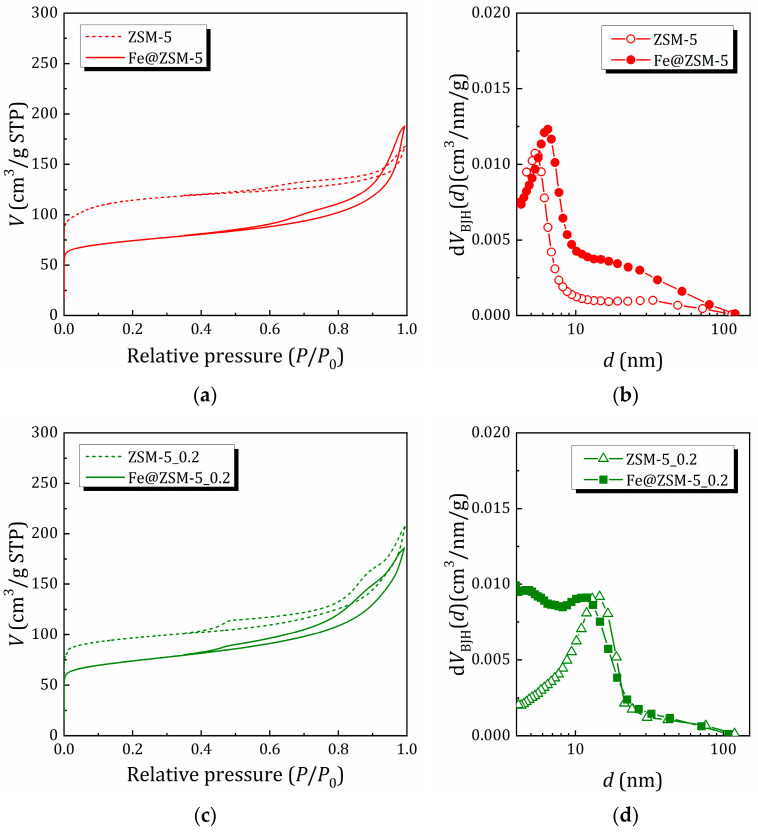

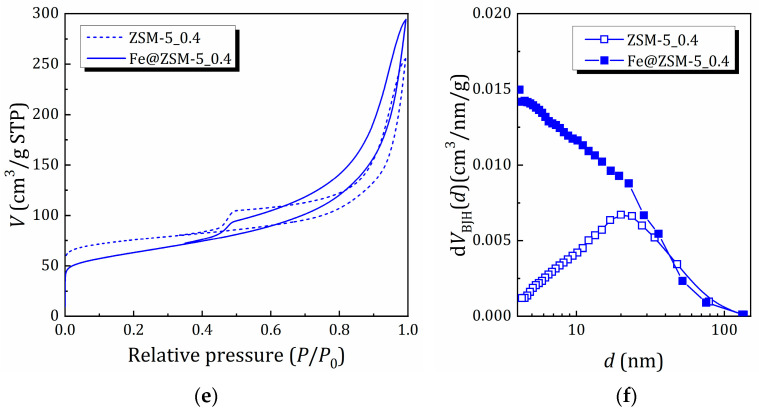
N_2_ adsorption/desorption isotherms (**a**,**c**,**e**) and BJH pore size distribution (**b**,**d**,**f**) for the zeolite matrices before and after Fe incorporation.

The N_2_ adsorption/desorption isotherms at 77 K for the samples before and after iron incorporation, as well as the pore size distribution evaluated using the BJH (Barrett, Joyner and Halenda) method, are shown in Figure 3. The surface area (*S*_BET_) estimated by the Brunauer–Emmett–Teller (BET) method, as well the BJH pore volume *V*_BJH_ are listed in Table 3. The textural properties of the zeolite matrices used as supports for iron species were analyzed in Ref. [40]; here, we have focused on the role of iron incorporation.

For the ZSM-5 sample with high microporosity, the introduction of iron results in a significant decrease in surface area and micropore volume (*V*_micro_); however, the total pore volume increases due to the appearance of larger mesopores. For other zeolite matrices pretreated in NaOH solution to introduce mesoporosity, iron incorporation also decreases the contribution of micropores to the surface area and pore volume, but to a lesser extent. It can also be noted that the ratio of mesopore volume *(V*_meso_) to micropore volume (*V*_micro_) increases (more dramatically for Fe@ZSM-5_0.4), which is due to the appearance of smaller mesopores. This allows us to suggest that (i) iron species block micropores and (ii) in the matrix with pre-introduced mesoporosity, these species are formed inside the mesopores, as also indicated by XPS analysis.

### 2.4. Magnetic Properties

**Figure 4:**
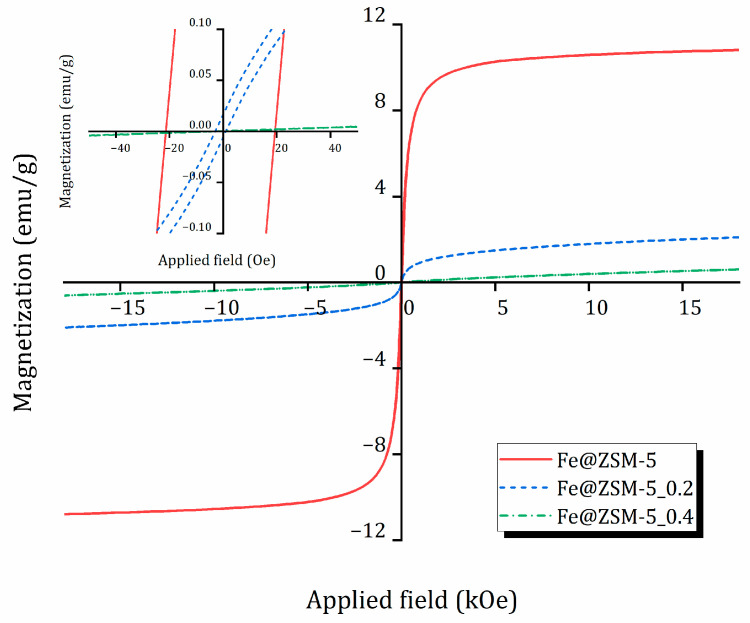
Magnetization hysteresis loop for studied composites at 300 K.

The magnetic properties of the iron-containing composites—saturation magnetization (*M_s_*) in the field range 0–1.8 kOe and coercive force (*H_c_*)—were measured at room temperature using vibrating-sample magnetometry (VSM). Magnetization curves are shown in Figure 4, and key parameters are summarized in Table 4.

Sample Fe@ZSM-5 exhibits a hysteresis loop typical of superparamagnetic magnetite nanoparticles (Fe_3_O_4_). The magnitude of the coercive force is ≈ 23 Oe (see also Figure S3, Supplementary Materials). Its specific saturation magnetization is 11 emu/g, which is considerably lower than that of bulk Fe_3_O_4_ (~90–100 emu/g) but comparable with other reported Fe_3_O_4_/zeolite composites [59]. This reduction is primarily attributed to the relatively low magnetite content and nanoscale particle size.

The magnetization curve of Fe@ZSM-5_0.2 reflects an intermediate behavior between paramagnetic and superparamagnetic states, suggesting the presence of additional iron-containing phases not detectable by XRD. Saturation is not reached even at 18 kOe. The estimated specific magnetization is approximately 2.5 emu/g, consistent with a decreased fraction of the magnetite phase, and the coercivity is about 4 Oe.

For the composite based on the most extensively desilicated matrix (Fe@ZSM-5_0.4), magnetic performance deteriorates sharply, which is attributed to partial oxidation of magnetite during synthesis or storage. This sample shows negligible superparamagnetic properties at room temperature (specific magnetization is only 0.7 emu/g) and a magnetization curve characteristic of paramagnetic materials.

### 2.5. Mössbauer Spectroscopy Studies

To identify the iron species in the obtained composites, ^57^Fe Mössbauer spectroscopy was applied. The experimental spectra of the three samples differ markedly from one another and exhibit distinct temperature dependent behavior.

**Figure 5:**
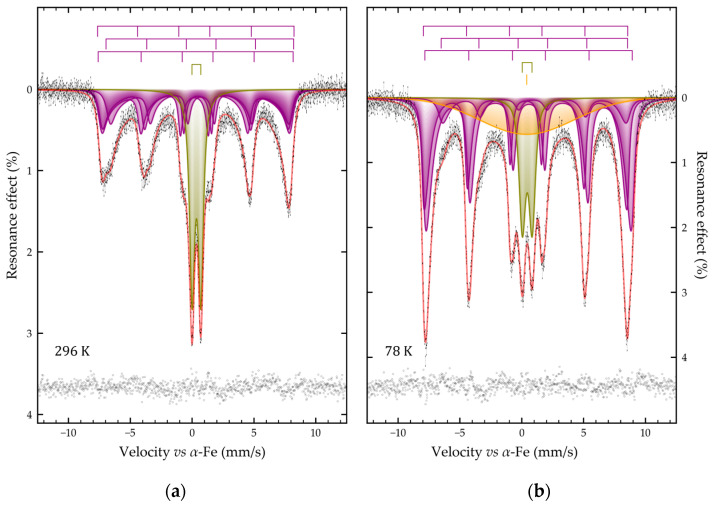
Mössbauer spectra of Fe@ZSM-5 sample obtained at 296 K (**a**) and 78 K (**b**). The size of the dashes corresponds to the error of the experimental points; the result of subtracting the model from the experimental spectrum is presented at the bottom of the figures.

As shown in Figure 5, the Mössbauer spectra of Fe@ZSM-5 sample (iron species supported on microporous parent ZSM-5) recorded at 296 K and 78 K consist of broadened distorted Zeeman sextets superimposed on paramagnetic doublets. The sextet lines are significantly broadened toward the center of the spectrum, with broadening being more pronounced at room temperature (Figure 5a). Upon cooling to 78 K, the relative intensity of the magnetic sextets increases substantially: at 296 K their integrated area is approximately one-third that of the paramagnetic doublet, whereas at 78 K it becomes ~1.5 times larger (Figure 5b). This spectral profile and its temperature evolution are typical of nanoscale magnetite-based materials undergoing superparamagnetic relaxation [60,61].

The room-temperature spectrum was satisfactorily fitted by the superposition of one symmetrical quadrupole doublet and three magnetically split sextets (Figure 5a). If the doublet profile is described by the pseudo-Voigt function, then the sextets were set in accordance with the many-state superparamagnetic relaxation model. In this case, all three sextets were interconnected by relaxation parameters (Table 5). To satisfactorily describe the experimental spectra of the Fe@ZSM-5 sample at 78 K the model described above had to be supplemented with a singlet with a very large width (Figure 3b, Table 3). This component clearly corresponds to isolated poorly crystallized iron-containing clusters with weakly expressed ferromagnetism localized presumably in zeolite voids (microporous zeolites with MFI topology are characterized by channels in size of 5.1 Å × 5.5 Å and 5.3 Å × 5.6 Å) or on the external zeolite surface. Obviously, at room temperature, the contribution of this component is small, possibly due to a weak bond with the zeolite matrix.

The obtained hyperfine parameters of the three sextets make it possible to attribute the materials to partially oxidized magnetite. Its composition can be expressed by the formula Fe_3-δ_O_4_ (δ > 0). The sextets can be attributed to Fe^3+^ iron ions in three crystallographic positions of the spinel structure (Fe^3+^)_Td_(Fe^2+^_1−3δ_Fe^3+^_1+2δ_#_δ_)_Oh_O_4_, where δ is the non-stoichiometry parameter of magnetite [62], # is a vacancy in the O_h_-sites [63,64]. The areas of these subspectra can be related by the following expression:

(1) $$S_{T_{d}}=\;\frac{{f\prime }_{T_{d}}}{{f\prime }_{O_{h}}}\frac{\Sigma S_{O_{h}}}{\left(2-\delta \right)}\;,$$

where $S_{T_{d}}$ is the relative area of a sextet with a lower isomeric shift (Table 5, subspectrum 1) corresponding to Fe^3+^ ions in tetrahedral positions of the spinel structure, $\Sigma S_{O_{h}}$ is the sum of the relative areas of sextets with a higher isomeric shift (Table 5, subspectra 2 and 3) from Fe^2+^ and Fe^3+^ ions in octahedral positions of the spinel structure [65], ${f\prime }_{T_{d}}$ and ${f\prime }_{O_{h}}$ are the probabilities of the Mössbauer effect (recoilless fractions) for Fe atoms in tetrahedral and octahedral positions, respectively. The ratio of recoilless fractions ${f\prime }_{T_{d}}/{f\prime }_{O_{h}}$ was assumed to be 0.98 and 0.94 for 78 and 296 K, respectively [66]. Since the ratio of ions of different charges in octahedral positions determines the average degree of oxidation of iron atoms, this affects the isomeric shift observed in the Mössbauer spectra [67]. It can be shown that the nonstoichiometry parameter (δ) of magnetite (Fe_3-δ_O_4_) is related to the area of partial spectra in octahedral positions ( $S_{O_{h}}$ ) and the corresponding isomeric shifts ( $\delta_{O_{h}}$ ) by the following expression [68,69]

(2) $$\delta =\frac{\Sigma (\delta_{2}-3\delta_{O_{h}}+2\delta_{3})S_{O_{h}}+(\delta_{2}-\delta_{3})S_{T_{d}}}{\Sigma (3\delta_{2}-\delta_{O_{h}}-2\delta_{3})S_{O_{h}}+3(\delta_{2}-\delta_{3})S_{T_{d}}}\;,$$

where $\delta_{2}$ = 1.33 ± 0.09 (or 1.16 ± 0.06) mm/s and $\delta_{3}$ = 0.49 ± 0.04 (or 0.37 ± 0.04) mm/s are isomeric shifts for Fe^2+^ and Fe^3+^ ions at 78 (or 296) K in an octahedral oxygen environment [67], $S_{T_{d}}$ is the relative area of the subspectrum in the tetrahedral position. $S_{T_{d}}$ and $S_{O_{h}}$ were calculated from the experimental spectra taking into account the above-mentioned ${f\prime }_{T_{d}}/{f\prime }_{O_{h}}$ ratio. The calculations performed according to Equation (2) using the data listed in Table 3 allow us to write the composition of ferromagnetic iron-containing particles as Fe_2.782(11)_O_4_ and Fe_2.777(5)_O_4_ for 296 and 78 K, respectively. Thus, the Fe@ZSM-5 sample should be considered as deeply oxidized magnetite.

The parameter α of the relaxation model for the sample Fe@ZSM-5 at 296 and 78 K was 3.81(11) and 10.95(25), respectively. This parameter is equal to the ratio of the anisotropy energy of the particle to the thermal energy:

(3) $$\alpha =\frac{KV}{k_{B}T}\;,$$

where *K* is the magnetic anisotropy constant, *V* is the volume of the magnetic domain, $k_{B}$ is the Boltzmann constant, and *T* is the temperature. Let us assume that *K* does not depend on temperature, but is an additive function depending on the composition of the material:

*K* = *K*(γ-Fe_2_O_3_)+*X*∙[*K*(Fe_3_O_4_) − *K*(γ-Fe_2_O_3_)], (4)

where *K*(Fe_3_O_4_) = 1.35 × 10^4^ J/m^3^ [70] and *K*(γ-Fe_2_O_3_) = 4.7 × 10^3^ J/m^3^ [71] constants of magnetic anisotropy for pure iron oxides, and *X* is the formal mole fraction of magnetite in the material, which is related to the non-stoichiometry parameter $\delta_{2}$ from Equation (2) by the following expression [62]:

(5) $$X=\frac{1-3\delta }{1+\delta }\;.$$

Then, expressing the particle volume *V* from Equation (3), we can estimate the size of the magnetic domains, for example, using a simplified model of spherical-shaped particles. The magnetic domain sizes calculated in this way for the Fe@ZSM-5 sample were 16.04(20) and 14.69(9) nm for 296 and 78 K, respectively. The reason for the apparent decrease in the detected particle sizes with decreasing temperature is the additional consideration of small particles that were paramagnetic at room temperature. This estimation suggests that the magnetic nanoparticles are formed on the zeolite surface.

**Figure 6:**
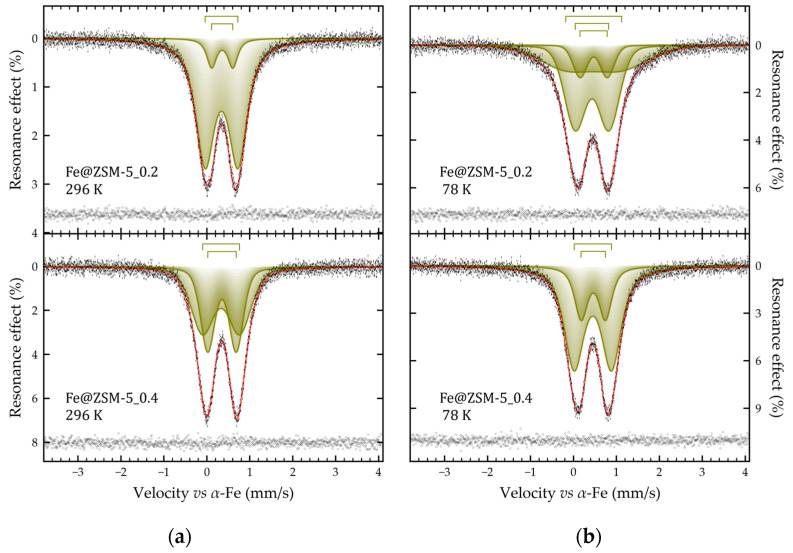
Mössbauer spectra of the Fe@ZSM-5_0.2 (**top**) and Fe@ZSM-5_0.4 (**bottom**) samples obtained at 296 K (**a**) and 78 K (**b**). The size of the dashes corresponds to the error of the experimental points; the result of subtracting the model from the experimental spectrum is presented at the bottom of the figures.

The experimental ^57^Fe Mössbauer spectra of Fe@ZSM-5_0.2 and Fe@ZSM-5_0.4 recorded at 78 and 296 K consist of well-resolved symmetric quadrupole doublets (Figure 6), whose intensity decreases significantly with increasing temperature. They can be fitted by a superposition of two (or three in the case of the sample Fe@ZSM-5_0.2 at 78 K) nested doublets with similar values of isomeric shifts (see Figure 6, Table 5). The values of isomeric shifts for the doublets of all samples correspond to Fe^3+^ ions in an octahedral oxygen environment [67] and are consistent with the literature data for its oxohydroxo compounds [72,73]. Based on the conditions of sample synthesis, it can be assumed that the spectra under consideration correspond to nanoscale particles of iron oxo- or oxohydroxo compounds in a superparamagnetic state with a blocking temperature significantly lower than the boiling point of nitrogen [61,74]. The low blocking temperature is indicated by the absence of temperature dependence of such hyperfine parameters as the quadrupole splitting and the resonance linewidths. The only exception is the spectrum at 78 K of the sample Fe@ZSM-5_0.2, for which it is necessary to introduce into the model an additional doublet with high values of quadrupole splitting and linewidth (Table 5). This doublet formally describes the incipient magnetic ordering with decreasing temperature.

Thus, based on the data obtained, it can be stated that all composite samples contain nanoscale iron- based clusters in the superparamagnetic state, but with markedly different blocking temperatures. Moreover, if for the Fe@ZSM-5 sample, the blocking temperature is higher than room temperature, then for Fe@ZSM-5_0.2 and Fe@ZSM-5_0.4 it is much lower than 78 K, and for and Fe@ZSM-5_0.4 this temperature is the lowest.

**Figure 7:**
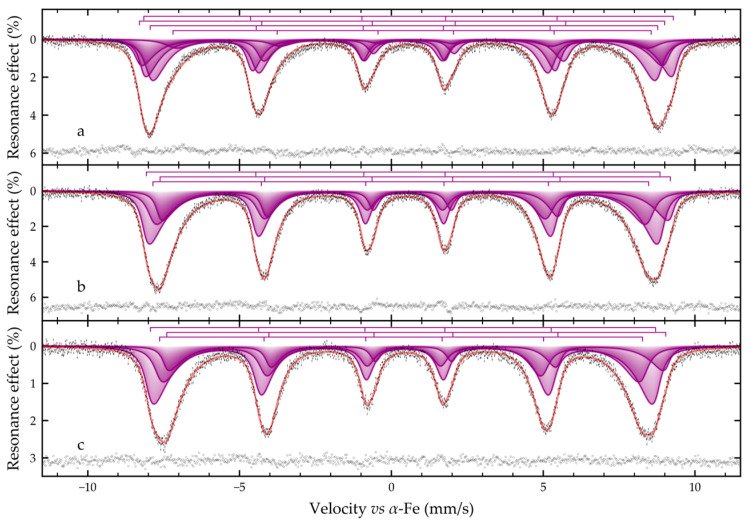
Mössbauer spectra of the (**a**) Fe@ZSM, (**b**) Fe@ZSM-5_0.2 and (**c**) Fe@ZSM-5_0.4 samples obtained at 5 K. The size of the dashes corresponds to the error of the experimental points; the result of subtracting the model from the experimental spectrum is presented at the bottom of the figures.

Indeed, when all samples are cooled to 5 K, their Mössbauer spectra become similar to each other and represent a superposition of Zeeman sextets (Figure 5). At the same time, despite the low temperature of spectral registration, their profile remains distorted by relaxation phenomena. The low-temperature spectrum of the Fe@ZSM-5 sample can be satisfactorily described by superposition of only four relaxation sextets (similar to the one described above), with associated relaxation parameters (Table 5). The presence of low-intensity resonance absorbances in the range of −6.5 and −3.0 mm/s on the spectrum profile (Figure 7a) is due to Fe^2+^ in octahedral positions, and is typical for low-temperature magnetite spectra [65,75,76]. In contrast to the described above spectrum of the Fe@ZSM-5 sample on the low-temperature experimental Mössbauer spectra of samples Fe@ZSM-5_0.2 and Fe@ZSM-5_0.4 these absorptions are not observed (Figure 7b,c). However, this may be due to the significantly larger width of the resonance lines for the discussed spectra. The spectra of these two samples can be satisfactorily described within the framework of a model as a superposition of three relaxation sextets (as indicated above), with associated relaxation parameters (Table 5). The calculated hyperfine parameters, such as isomeric shift and quadrupole shift for these samples vary little within the limits of experimental error, which indicates the uniform nature of iron-containing species. However, the widths of the resonance lines and the hyperfine magnetic fields of the sextets for the sample Fe@ZSM-5_0.4 differ markedly from similar parameters for the sample Fe@ZSM-5_0.2 (Table 5): the widths are larger, and the magnitudes of the magnetic splits are smaller. The reason for this phenomenon is the significantly smaller size of the magnetic domains for the sample Fe@ZSM-5_0.4 compared to the Fe@ZSM-5_0.2 sample. Similar observations can be made by comparing the corresponding parameters of these two samples with Fe@ZSM-5: the Fe@ZSM-5 sample differs somewhat in structure (at least in greater order) and in the large size of the magnetic domains (crystallites).

### 2.6. UV-Vis Diffuse Reflectance Spectroscopy Studies

**Figure 8:**
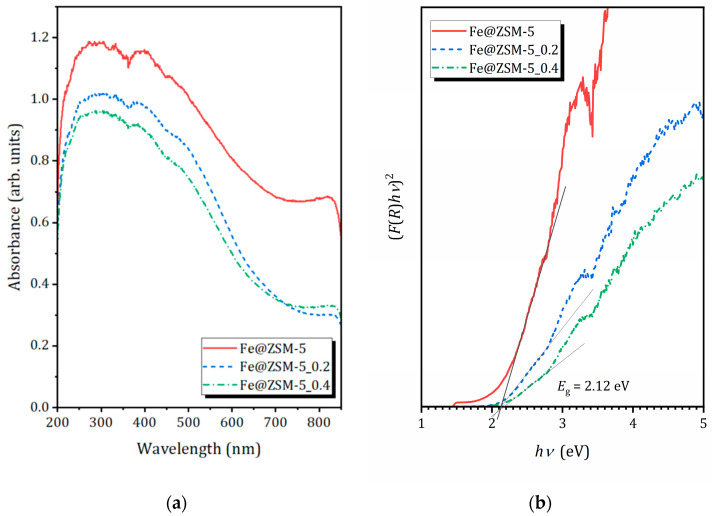
(**a**) UV-vis spectra of the composites; (**b**) (*F*(*R*)*hν*)^2^ versus photon energy for calculation of bandgap.

The optical properties of the prepared composites were characterized by UV-Vis DRS. The spectra of all samples are shown in Figure 8a. They exhibit similar overall profiles, with at least four main absorption bands, centered at approximately 220, 300, 390 and 450–490 nm. Deconvolution of the spectra are shown in Figure S4 (Supplementary Materials).

According to literature reports [77,78,79] the sub-band at 240 nm can be associated with isolated Fe^3+^ ions in tetrahedral coordination (charge-transfer transitions involving framework oxygen). The broader band centered at about 370 nm is typically assigned to Fe^3+^ ions belonging to small oligonuclear Fe_x_O_y_ clusters; whereas the intense absorption extending from ~450 to 490 nm and beyond is characteristic of larger magnetite Fe_3_O_4_ nanoparticles. The contribution from Fe^2+^ d–d transitions is expected above 800 nm and thus lies outside the measured range. All these features are clearly observed in the studied composites, with the relative intensities varying between samples in full agreement with Mössbauer spectroscopy results.

In all composites, Fe_x_O_y_ clusters and Fe_3_O_4_ nanoparticles predominate, while a minor fraction of isolated Fe^3+^ ions are also present. The latter may partially isomorphically substitute Al^3+^ in the zeolite framework. The contribution of crystalline hematite (α-Fe_2_O_3_) nanoparticles or other bulk iron oxide phases is negligible, consistent with the absence of corresponding reflections in the XRD. Nevertheless, the presence of small amount of various iron-containing nanospecies likely causes partial blockage of the MFI micropores, contributing to the observed reduction in specific surface area.

Band gap energies (*E*_g_), were determined from the transformed diffuse reflectance spectra of the studied composites using the Kubelka–Munk function. The data were plotted as (*F(R*)*hν*)^2^ = *f*(*hν*), where *F*(*R*) = (1 − *R*)^2^/2*R* is the Kubelka–Munk expression of a reflection coefficient R. The direct bandgap value *E*_g_ was estimated from the point of intersection of the linear fit of the Kubelka–Munk graphs with abscise axis [80]. As can be seen from Figure 8b, all the samples show the same energy bandgap of 2.12 eV. This value confirms that these materials are photoactive not only under ultraviolet irradiation but also throughout a significant portion of the visible spectrum, making them promising for solar-driven photocatalytic applications.

## 3. Materials and Methods

### 3.1. Synthesis

#### 3.1.1. Preparation of Mesoporous ZSM-5 Matrices

NH_4_-ZSM-5 zeolite (MFI framework type, nominal Si/Al = 15, product CBV 3024E) supplied by Zeolyst International (Kansas City, KS, USA) was used as the parent material. Mesoporous ZSM-5 matrices were prepared by alkaline treatment (desilication) of the parent zeolite in aqueous NaOH solution (>99.0%, supplied by Vekton, Saint Petersburg, Russia). In a typical procedure, the zeolite was suspended in 0.2 or 0.4 M NaOH solution and stirred (350 rpm) at 65 °C for 120 min. The suspension was then centrifuged (10–15 min), washed repeatedly with distilled water until the washings reached neutral pH, and dried overnight at 100 °C. The resulting materials are denoted as ZSM-5_0.2 and ZSM-5_0.4, where the number indicates the concentration of NaOH used for desilication.

#### 3.1.2. Synthesis of Magnetite–Zeolite Composites

Magnetite–zeolite composites were prepared by co-precipitation of Fe^2+^ and Fe^3+^ ions in the presence of the zeolite matrix under oxygen-free conditions. The following reagents were used: FeCl_3_·6H_2_O (≥99%), FeSO_4_·7H_2_O (≥99%), NaOH (reagent grade), CH_2_Cl_2_ (≥99.8%), and deionized water. All chemicals are supplied by Vekton (Saint Petersburg, Russia).

In a typical synthesis, 0.50 g of zeolite (parent ZSM-5 or mesoporous ZSM-5_0.2/ZSM-5_0.4) was dispersed in 10 mL of deionized water previously deoxygenated by bubbling with argon for at least 30 min. Subsequently, 0.30 g of FeCl_3_·6H_2_O (corresponding to [Fe^3+^] = 0.111 M in the final reaction mixture) and 0.24 g of FeSO_4_·7H_2_O ([Fe^2+^] = 0.086 M in the final reaction mixture) were added. The suspension was stirred vigorously, evacuated using a water-jet pump, and maintained under vacuum with continuous stirring for 15 min to promote impregnation of iron species into the zeolite pores. Thereafter, a deoxygenated 1 M NaOH solution was added dropwise until the pH reached 10–12 (monitored with universal indicator paper). The mixture was then stirred vigorously for 1 h at room temperature under an argon atmosphere. All solutions and water used throughout the synthesis were pre-degassed with argon to prevent premature oxidation of Fe^2+^.

The resulting dark precipitate was collected by filtration, washed thoroughly with deionized water followed by methylene chloride to remove residual chloride and sulfate ions, and dried under reduced pressure on a rotary evaporator (bath temperature 60 °C, 40 mbar) for 1 h. The obtained composites were stored under argon in sealed vials to prevent oxidation of the magnetite phase.

The samples are designated as Fe@ZSM-5 (iron supported on parent microporous ZSM-5), Fe@ZSM-5_0.2, and Fe@ZSM-5_0.4 (iron supported on matrices treated with 0.2 M and 0.4 M NaOH, respectively).

### 3.2. Characterization

XRD patterns were recorded on a Rigaku Miniflex II benchtop diffractometer (Rigaku Corporation, Tokyo, Japan) using Cu K_α_ radiation (λ = 1.5406 Å). Data were collected in the 2θ range of 3–60° with a step width of 0.02° and a scan rate of 2° min^−1^. Elemental composition was determined by EDXRF under vacuum using a Shimadzu EDX 800 HS apparatus (Shimadzu Corporation, Kyoto, Japan). SEM-EDX studies were performed using a Zeiss Merlin field-emission scanning electron microscope (Carl Zeiss Microscopy GmbH, Oberkochen, Germany) equipped with an Oxford Instruments INCAx-act EDX console.

XPS spectra were obtained using a Thermo Fisher Scientific Escalab 250Xi spectrometer (Thermo Fisher Scientific, Waltham, MA, USA) with non-monochromatic Al Kα radiation (photon energy 1486.6 eV).

N_2_ adsorption/desorption isotherms were obtained at 77 K using the Quadrasorb SI 2SI-MP-20 equipment (Quantachrome Instruments, Boynton Beach, FL, USA). Before analysis, the samples were outgassed under vacuum in a FLOVAC Degasser FVD-3 degasser (Anton Paar GmbH, Graz, Austria) for 3 h at 300 °C.

Magnetic properties were measured at 300 K using a Lake Shore Cryotronics Inc. (Westerville, Ohio, USA) Model 7410 vibrating-sample magnetometer (VSM). Hysteresis loops were recorded by sweeping the applied magnetic field from −18 to +18 kOe at a frequency of 77 Hz.

Diffuse reflectance UV–Vis spectra (DRS) were recorded on a Persee T8DCS spectrophotometer (Auburn, CA, USA) equipped with an SI19-1 integrating sphere. Spectra were collected over the wavelength range of 190–900 nm using BaSO_4_ as a reference.

^57^Fe Mössbauer absorption spectra were measured in transmission geometry with a moving source and triangular velocity reference signals on an express Mössbauer spectrometer MS1104EM (CJSC Kordon, Rostov-on-Don, Russia). Spectra were accumulated at 296(3) K, 77.7(0.3) K and 5.000(3) K using a Cryogen-free closed cycle cryostat for Mössbauer spectroscopy CFPR-221-MESS (LLC Kriogennye Pribory, Moscow, Russia). The source of γ-radiation was ^57^Co in a matrix of metallic rhodium with an activity of approximately 10 mCi (Cyclotron Co., Ltd., Obninsk, Russia), maintained at room temperature during measurements. Velocity calibration was performed using a standard α-Fe foil absorber. The signal-to-noise ratio of the accumulated spectra did not exceed 2%. High-resolution spectra (1024 channels) were processed and fitted using SpectRelax 4.1 software (Lomonosov Moscow State University, Moscow, Russia) [81]. Values of isomer shifts are reported relative to α-Fe at 296 K.

## 4. Conclusions

The influence of tunable hierarchical porosity of ZSM-5 zeolite on the composition and properties of iron-containing species formed during the synthesis of magnetite supported on the zeolite matrix has been studied. Magnetite is present in all samples; however, the particle size and stoichiometry are strongly governed by the extent of post-synthetic desilication. The generated mesoporosity in the ZSM-5 matrix plays a decisive role in the nature of the supported iron species: mild alkaline treatment promotes the formation of nanocrystalline, partially oxidized magnetite mainly located on the external surface of the zeolite, whereas strong desilication leads to the formation of ultra-small, highly dispersed Fe(III) oxide/hydroxide clusters confined within mesopores. This structural evolution results in a gradual transition from superparamagnetic behavior at room temperature to paramagnetic, accompanied by a sharp decrease in saturation magnetization.

Despite these differences, all composites exhibit identical optical band gaps of ~2.12 eV, ensuring efficient visible-light harvesting. The obtained materials combine high adsorption capacity and magnetic separability, making the microporous composite (Fe@ZSM-5) particularly promising as a reusable visible-light photocatalyst with excellent mass transfer properties due to the developed mesoporosity.

The present study highlights the important role of controlled mesoporosity generation in the support for tuning the dispersion of the active phase and provides a rational basis for the design of advanced magnetite–zeolite photocatalysts for solar-driven water purification. Thus, the studied composites have great potential for use in additive technologies combining photocatalysis, high kinetics, and magnetic separation. However, further optimization efforts are needed, as increasing porosity (important for transport properties) simultaneously leads to greater dispersion and smaller magnetite particles, thereby reducing magnetic response.

## Figures and Tables

**Table 1:** Chemical composition (atomic ratio) and Fe weight % for the studied samples as determined from EDXRF.

Sample	Si/Al	Fe/Al	Fe (wt%)
ZSM-5 *	10.15		
ZSM-5_0.2 *	7.77		
ZSM-5_0.4 *	4.96		
Fe@ZSM-5	11.1	11.1	26.7
Fe@ZSM-5_0.2	7.1	5.9	22.6
Fe@ZSM-5_0.4	6.7	11.3	34.9

* data from Ref. [40].

**Table 2:** Fe 2*p* and O 1*s* XPS valence analysis results (total areas in %) of the Fe@zeolite composites; the corresponding binding energies (*E*_b_) are provided.

Sample	Fe		O	
	Fe^2+^	Fe^3+^	O_Fe_	O_Z_
	*E*_b_ = 710.5 eV	*E*_b_ = 712.6 eV	*E*_b_ = 530.1 eV	*E*_b_ = 532.1 eV
Fe@ZSM-5	37	63	79	21
Fe@ZSM-5_0.2	39	61	75	25
Fe@ZSM-5_0.4	40	60	74	26

**Table 3:** Textural properties of the samples before and after Fe incorporation.

Sample	*S*_BET_ (m^2^/g)	*V*_BJH_ (cm^3^/g)	*V*_meso_ (cm^3^/g)	*V*_micro_ (cm^3^/g)	*V*_meso_/*V*_micro_	*D*_BJH_ (nm)
ZSM-5	429	0.259	0.084	0.168	0.5	5
Fe@ZSM-5	281	0.291	0.190	0.109	1.7	6.5/15
ZSM-5_0.2	374	0.320	0.167	0.139	1.2	13
Fe@ZSM-5_0.2	277	0.287	0.184	0.108	1.7	>15
ZSM-5_0.2	284	0.395	0.262	0.111	2.4	23
Fe@ZSM-5_0.2	227	0.455	0.368	0.088	4.2	>23

**Table 4:** Magnetic parameters of the studied composites (at 300 K).

Sample	*M_s_* (emu/g)	*H_c_* (Oe)
Fe@ZSM-5	11	23
Fe@ZSM-5_0.2	~2.5	4
Fe@ZSM-5_0.4	0.7	-

**Table 5:** Hyperfine parameters of the Mössbauer spectra at different temperatures for the studied samples: δ is the isomer shift, ε {Δ = 2ε} is the quadrupole splitting, Γ_exp_ is the line width, H_eff_ is the hyperfine magnetic field, and S is the relative area of the subspectrum.

Sample	Temperature (K)	No.	δ (mm/s)	ε (mm/s)	Δ (mm/s)	Γ_exp_ (mm/s)	H_eff_ (kOe)	S (%)
Fe@ZSM-5	296(3)	1	0.250(21)	0.060(21)		0.300(23)	492.9(1.4)	27.82(13)
		2	0.683(20)	−0.054(13)		0.36(4)	470.7(1.9)	22.7(2.3)
		3	0.372(25)	−0.084(23)		0.26(3)	489.0(2.0)	23.9(2.3)
		4	0.36(1)		0.71(1)	0.50(1)		25.6(5)
	77.7(3)	1	0.33(1)	0.00(1)		0.36(1)	512.5(4)	25.2(1.5)
		2	0.968(29)	0.059(16)		0.70(8)	467.6(2.0)	8.1(1.0)
		3	0.57(1)	−0.01(1)		0.497(11)	520.5(4)	35.7(2.3)
		4	0.45(1)		0.79(1)	0.63(1)		12.2(1.1)
		5	0.41(4)			9.0(4)		19(5)
	5.000(3)	1	0.49(1)	0.08(1)		0.400(21)	540.7(7)	30(3)
		2	0.55(1)	−0.192(13)		0.464(29)	536.4(8)	22.6(2.7)
		3	0.41(1)	0.01(1)		0.520(23)	518.2(8)	37(4)
		4	0.74(4)	−0.059(16)		0.77(10)	488.3(2.3)	10.4(2.2)
Fe@ZSM-5_0.2	296(3)	1	0.36(1)		0.493(17)	0.27(6)		11(6)
		2	0.34(1)		0.764(22)	0.52(1)		89(6)
	77.7(3)	1	0.47(1)		0.63(3)	0.39(6)		13(8)
		2	0.44(1)		0.777(20)	0.599(26)		52(7)
		3	0.475(12)		1.30(29)	1.49(17)		35(9)
	5.000(3)	1	0.42(1)	−0.02(1)		0.435(17)	524.5(5)	45(4)
		2	0.74(1)	0.05(1)		0.362(20)	521.0(4)	22.9(2.4)
		3	0.38(1)	−0.07(1)		0.541(25)	506.3(1.1)	32(3)
Fe@ZSM-5_0.4	296(3)	1	0.35(1)		0.66(1)	0.40(20)		44(8)
		2	0.33(1)		0.86(3)	0.649(25)		56(8)
	77.7(3)	1	0.46(1)		0.56(3)	0.37(6)		26(17)
		2	0.45(1)		0.87(6)	0.549(14)		74(17)
	5.000(3)	1	0.40(1)	−0.03(1)		0.531(15)	514.8(5)	51(3)
		2	0.767(10)	0.04(1)		0.399(23)	509.2(4)	21.4(2.5)
		3	0.350(11)	−0.05(1)		0.548(27)	492.4(1.0)	27.3(2.7)

## Data Availability

The original contributions presented in this study are included in the article/Supplementary Materials. Further inquiries can be directed to the corresponding author(s).

## Supplementary Materials

The following supporting information can be downloaded at: https://www.mdpi.com/article/10.3390/molecules31010089/s1, Figure S1: SEM images for the studied composites (on the right) and parent zeolite matrices (on the left); Figure S2: Element distribution maps in the studied Fe@zeolite composites according to SEM-EDX; Figure S3: Magnetization hysteresis loop for studied composites; Figure S4: Decomposition of UV-Vis spectra (on the left); (*F*(*R*)*hν*)^2^ versus photon energy for calculation of bandgap energies for the studied composites (on the right).

## Abbreviations

The following abbreviations are used in this manuscript:

BET	Brunauer–Emmett–Teller (method)
BJH	Barrett–Joyner–Halenda (method)
DRS	Diffuse reflectance spectroscopy
EDX	Energy-dispersive X-ray (spectroscopy)
EDXRF	Energy dispersive X-ray fluorescence spectroscopy
SEM	Scanning electron microscopy
VSM	Vibrating-sample magnetometry
UV–Vis	Ultraviolet–visible spectroscopy
XRD	X-ray diffraction
ZSM-5	Zeolite Socony Mobil-5
