# Supplementary material for: Synthesis and Magnetic and Optical Properties of Novel Fe@ZSM-5 Composites
Source: Molecules. 2025 Dec 25;31(1):89. doi: 10.3390/molecules31010089 (PMC12786709; doi:10.3390/molecules31010089)
Supplement: Supplementary file 1 [file molecules-31-00089-s001.zip › molecules-4049135-supplementary.pdf]

SUPPLEMENTARY MATERIALS

Synthesis and Magnetic and Optical Properties of Novel Fe@ZSM-5 Composites

Irina A. Zvereva [1], Denis A. Pankratov [2,3], Elena G. Zemstova [1], Vladimir K. Kudymov [1], Azamat Samadov [1], Sergey A. Kurnosenko [1], Sergey O. Kirichenko [4], Marina G. Shelyapina [5,*] and Vitalii Petranovskii [6]

[1] Institute of Chemistry, Saint Petersburg State University, 7/9 Universitetskaya Embankment, Saint Petersburg 199034, Russia; irina.zvereva@spbu.ru (I.A.Z.); e.zemtsova@spbu.ru (E.G.Z.); v.k.kudymov@spbu.ru (V.K.K.); st088712@student.spbu.ru (A.S.); s.kurnosenko@spbu.ru (S.A.K.)

[2] Department of Chemistry, Lomonosov Moscow State University, Leninskie Gory 1/3, Moscow 119991, Russia; pankratov@radio.chem.msu.ru

[3] Moscow Center for Advanced Studies, 20, Kulakova Str., Moscow 123592, Russia

[4] Centre for Innovative Technologies of Composite Nanomaterials, Saint Petersburg State University,
7/9 Universitetskaya Embankment, Saint Petersburg 199034, Russia; sergey.kirichenko@spbu.ru

[5] Department of Nuclear Physics Research Methods, Faculty of Physics, Saint Petersburg State University,
7/9 Universitetskaya Embankment, Saint Petersburg 199034, Russia

[6] Centro de Nanociencias y Nanotecnología, Universidad Nacional Autónoma de México, Ensenada 22800, BC, Mexico; vitalii@ens.cnyn.unam.mx

* Correspondence: marina.shelyapina@spbu.ru

ZSM-5 | Fe@ZSM-5

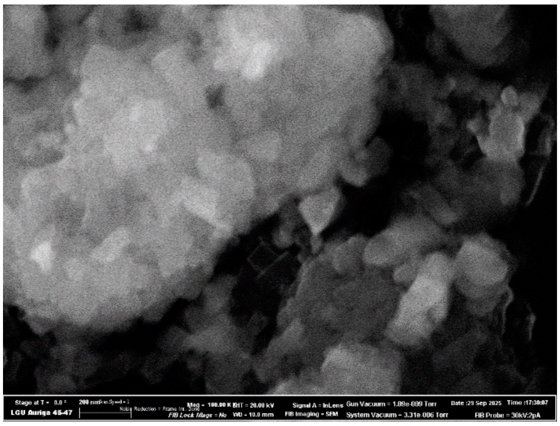

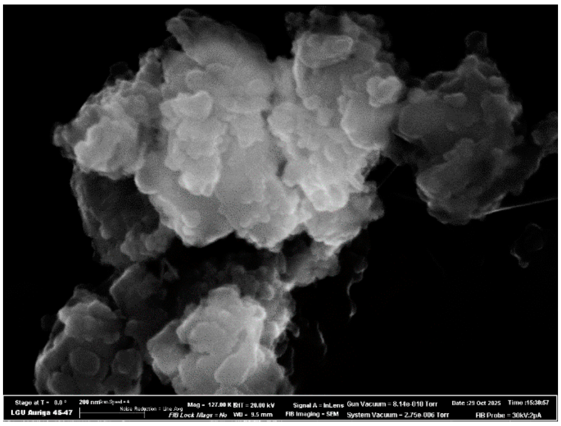

ZSM-5_0.2 | Fe@ZSM-5_0.2

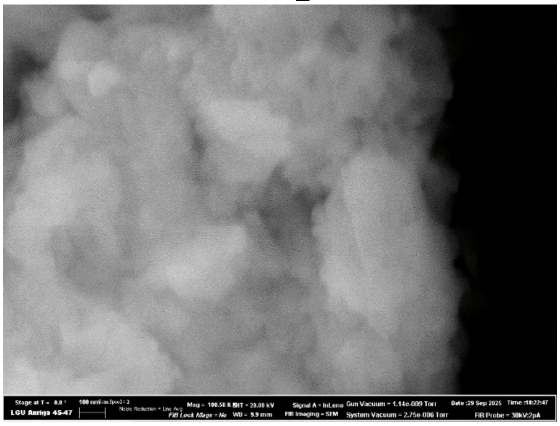

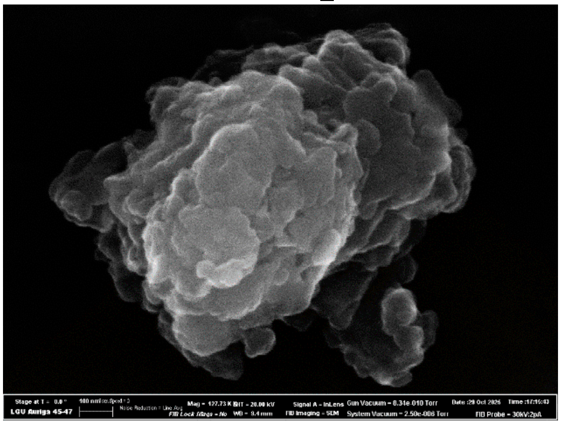

ZSM-5_0.4 | Fe@ZSM-5_0.4

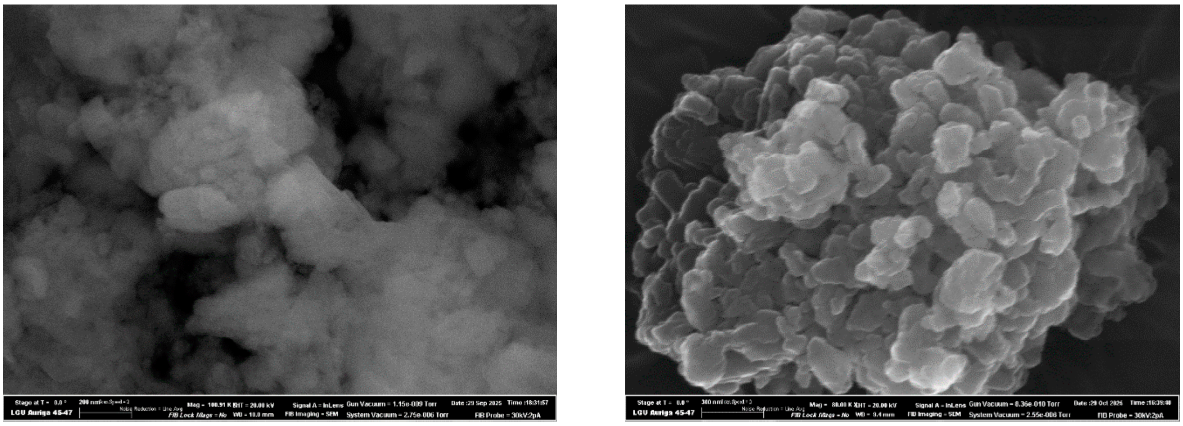

Figure S1. SEM images for the studied composites (on the right) and parent zeolite matrices (on the left).

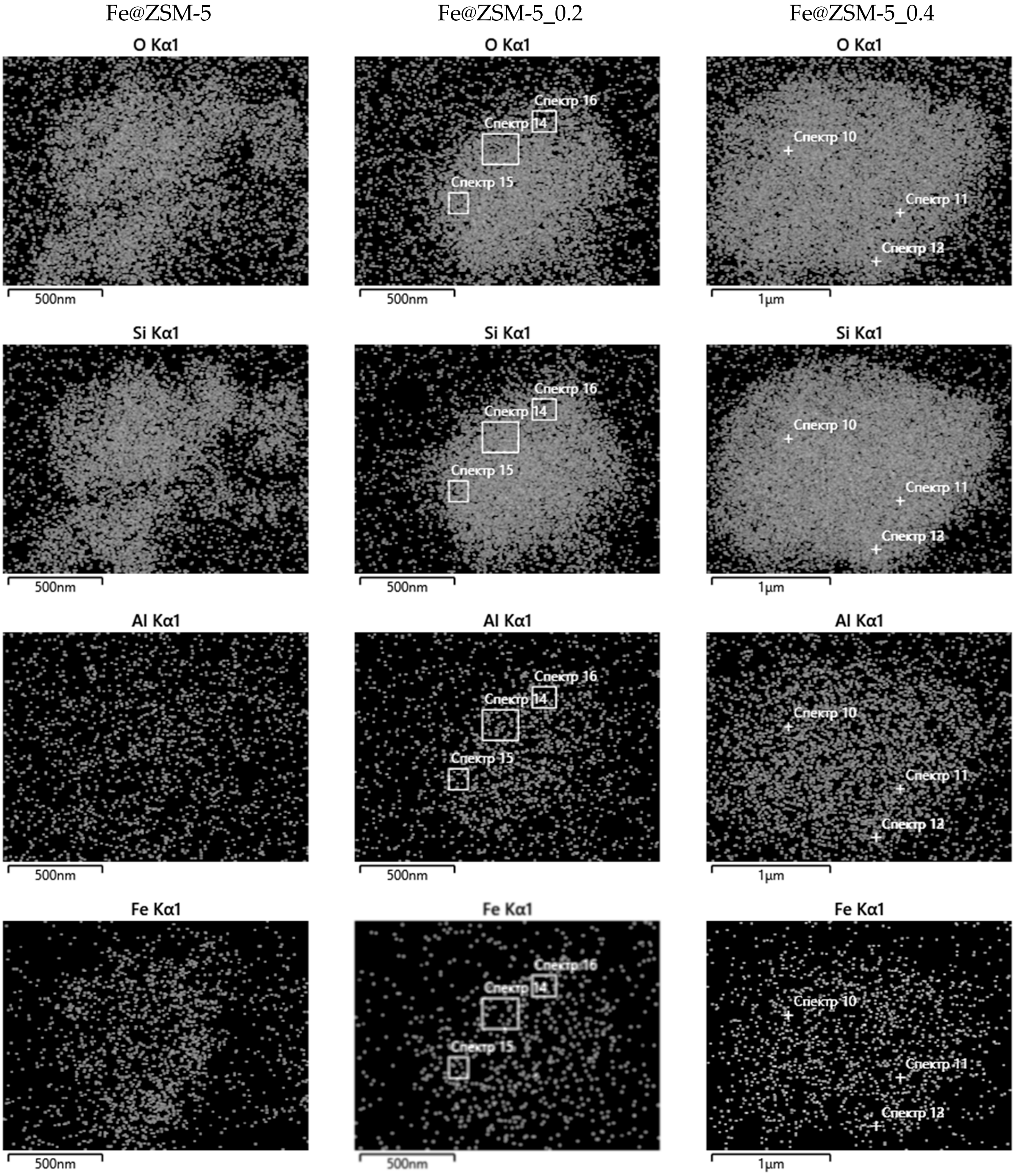

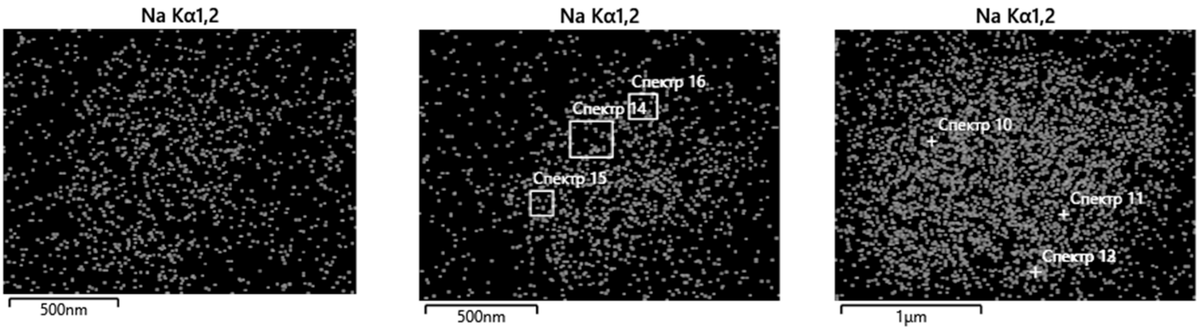

Figure S2. Element distribution maps in the studied Fe@zeolite composites according to SEM-EDX.

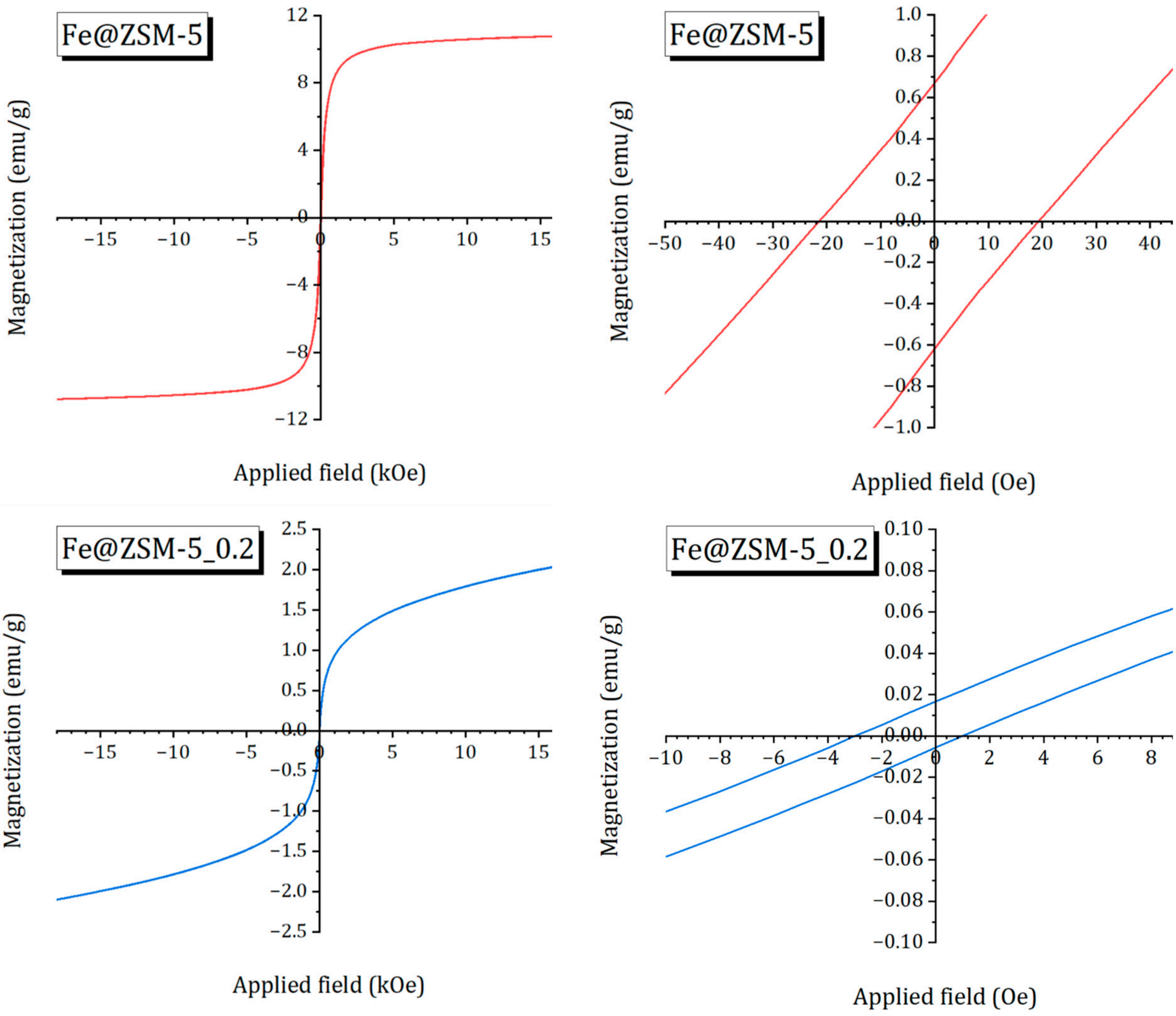

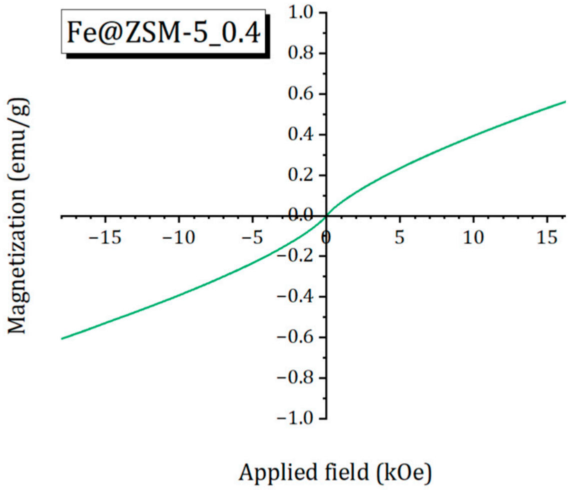

Figure S3. Magnetization hysteresis loop for studied composites.

Fe@ZSM-5

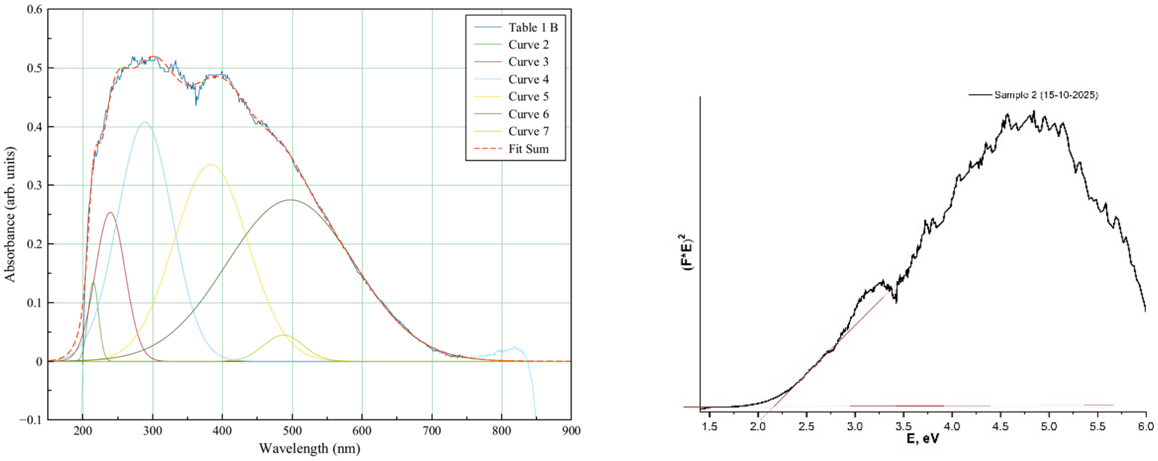

Fe@ZSM-5_0.2

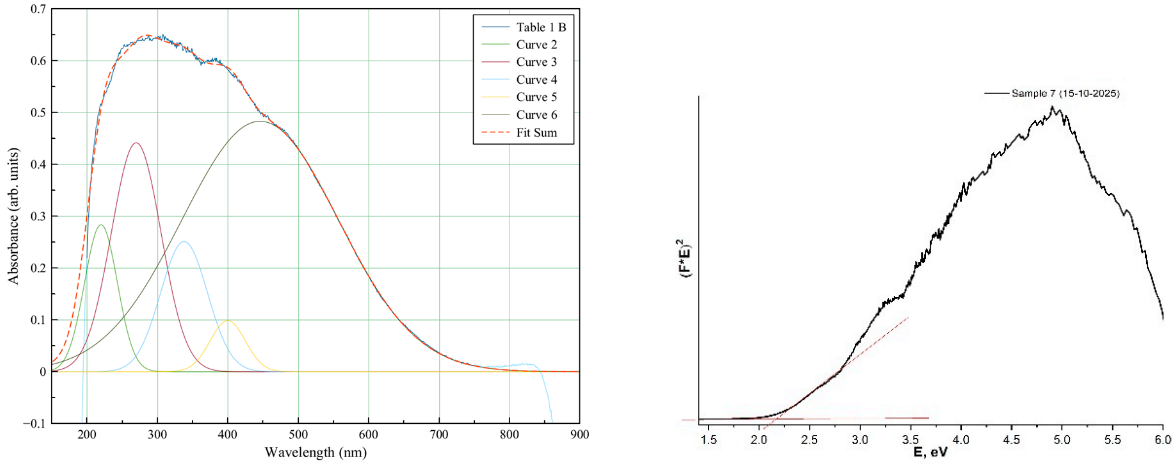

Fe@ZSM-5_0.4

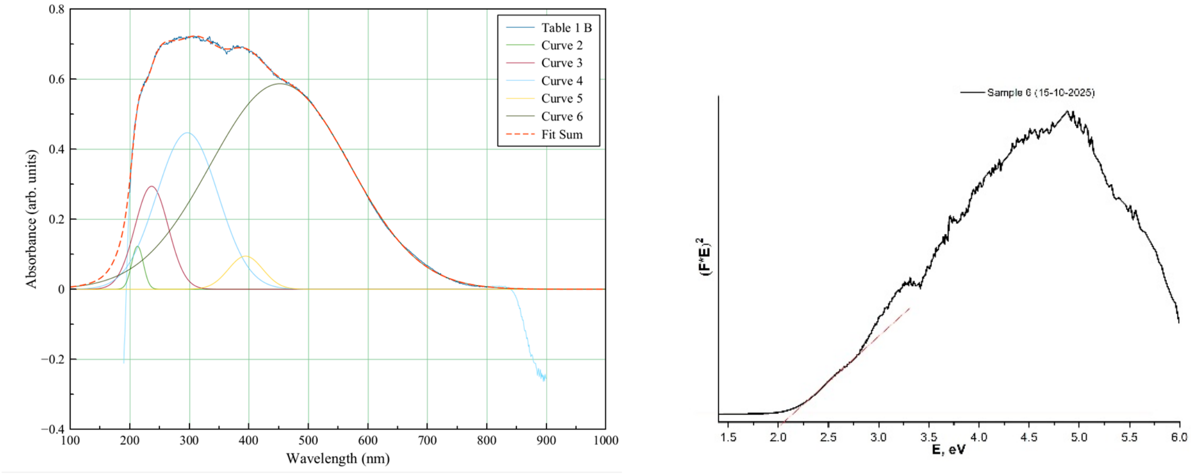

Figure S4. Decomposition of UV-Vis spectra (on the left); $(F(R)h\nu)^2$ versus photon energy for calculation of bandgap energies for the studied composites (on the right).
